# The role and importance of auxiliary tests in differential diagnosis in patients with mildly high basal 17-OH-progesterone levels in the evaluation of hirsutism

**DOI:** 10.3906/sag-2004-263

**Published:** 2020-12-17

**Authors:** Taner DEMİRCİ, Hasret CENGİZ, Ceyhun VARIM, Sedat ÇETİN

**Affiliations:** 1 Department of Endocrinology and Metabolism, Sakarya University Research and Education Hospital, Sakarya Turkey; 2 Department of Internal Medicine, Sakarya University Medicine Faculty, Sakarya Turkey

**Keywords:** Late-onset congenital adrenal hyperplasia (LOCAH), polycystic ovary syndrome (PCOS), 17-OH-progesterone, dehydroepiandrosterone sulphate (DHEAS), idiopathic hyperandrogenism (IH)

## Abstract

**Background/aim:**

In the differential diagnosis of hirsutism, early follicular basal 17-OH-progesterone levels sometimes overlap with the diagnosis of late onset congenital adrenal hyperplasia (LOCAH) and other causes of hyperandrogenism. This study aims to investigate the role of some common tests and clinical findings in differential diagnosis in such cases.

**Materials and methods:**

One hundred seventy-five female patients with hirsutism and mildly high initial 17-OH-progesterone levels (2-10 ng/mL) were included in the study. The cases were divided into three groups according to their diagnosis: LOCAH (n = 16, mean age = 26.1 ± 6.9), polycystic ovary syndrome (PCOS) (n = 122, mean age = 23.9 ± 5.1), and intracranial hypertension (IH) (n = 37, mean age = 25.2 ± 7.3). Clinical signs and symptoms, such as menstrual irregularity and hirsutism score, and hormone levels including total testosterone and dehydroepiandrosterone sulfate (DHEAS), were compared between the groups.

**Results:**

There was no difference between the groups with PCOS, LOCAH, and IH for total testosterone level results (P = 0.461). The DHEAS level was higher in the PCOS group than in the LOCAH group (449.6 ± 151.14 vs. 360.31 ± 152.40, P = 0.044). While there was no difference between the PCOS and LOCAH groups in terms of menstrual irregularity (P = 0.316), the hirsutism score for IH was significantly lower than those of PCOS and LOCAH (9.2 vs. 12.2 and 11.1, respectively; P < 0.001). Basal 17-OH-progesterone levels were higher in the LOCAH group than in the other groups (P = 0.016).

**Conclusion:**

While DHEAS level was lower in LOCAH than in PCOS, it was not different from that in IH. While the severity of hirsutism was higher in LOCAH than in IH, it was not different from that in PCOS. Menstrual irregularity was similar between PCOS and LOCAH. According to these results, although the auxiliary tests and clinical findings for the diagnosis of LOCAH contribute to the clinical interpretation, they are not superior to the 17-OH-progesterone level for diagnosis.

## 1. Introduction

Hirsutism is defined as male pattern hair growth in women. It affects an average of 5%–10% of women of reproductive age [1]. This situation can be a source of emotional stress that can affect daily life [2,3]. Hirsutism is a result of the interaction between circulating serum androgens and the sensitivity of hair follicles to these hormones. Increased serum androgen concentrations are defined as hyperandrogenism, which can cause hirsutism, acne, androgenic alopecia, and even virilization. The excessive growth of terminal hair in women in a male-like pattern is the most common clinical diagnostic finding of hyperandrogenism [4,5]. Although polycystic ovary syndrome (PCOS) constitutes 75%–80% of cases, there are many underlying causes of etiology in patients with hirsutism [6]. These causes can be serious clinical conditions such as androgen-secreting tumors and ovarian hyperthecosis, as well as Cushing syndrome, hyperprolactinemia, thyroid disorders, HAIR-AN syndrome, late-onset congenital adrenal hyperplasia (LOCAH), and idiopathic hyperandrogenism (IH) [7,8]. In the differential diagnosis, along with menstrual cycle pattern, laboratory methods are used, including measuring levels of human chorionic gonadotropin (hCG), prolactin, follicle-stimulating hormone (FSH), thyroid-stimulating hormone (TSH), and early morning 17-hydroxyprogesterone (performed around 8:00 AM). Imaging methods may be used when further investigation is required.

LOCAH (or nonclassical congenital adrenal hyperplasia) accounts for less than 5% of all causes of hirsutism. However, 60% of adult women diagnosed with LOCAH also have hirsutism. More than 90% of all congenital adrenal hyperplasia cases have a defect in the conversion of 17-hydroxyprogesterone to 11-deoxycortisol. The enzyme that performs this reaction is 21-hydroxylase, and the activity level of this enzyme is 20%–50% in LOCAH [9–12]. Measuring 17-hydroxyprogesterone concentration, the substrate of the missing enzyme, is the screening method used in diagnosis. For the first screening, if the woman has regular menstrual cycles, the serum samples should be taken in the morning (07:30 to 8:00) for 17-hydroxyprogesterone concentration during the follicular phase of the menstrual cycle. For women with irregular menses, samples can be drawn on a random day. Generally, a basal 17-hydroxyprogesterone value greater than 2 ng/mL (6 nmol/L) is sufficient for LOCAH diagnosis; however, the threshold value has been accepted as >10 ng/mL for definitive laboratory diagnosis. For moderate elevations in the 2–10 ng/mL range, the ACTH stimulation test (high dose, 250 mcg) is used for confirmation. Cases with 17-OH-progesterone levels higher than 10 ng/mL in response to the ACTH stimulation test are considered LOCAH [13]. Although the use of a genetic test is not necessary for the initial test, it should be used for genetic counseling before conception in the case of borderline laboratory values [9,11].

In this study, the importance of auxiliary laboratory tests and some clinical findings in the differential diagnosis of LOCAH was investigated in hirsute women whose basal 17-hydroxyprogesterone level was found to be slightly high.

## 2. Materials and methods

### 2.1 Subjects

This is a retrospective study in which data from July 2017 to December 2019 were scanned. One hundred and seventy-five female patients of reproductive age admitted to the endocrinology outpatient clinic due to increased hairiness and whose baseline 17-OH-progesterone level was found to be high at the limit were included in the study. The mean (±SD) age of the patients included in the study was 24.4 (±5.8). The age range was 18–48. Women under the age of 18 and postmenopausal women were excluded from the study. Demographic data, menstrual irregularity, and infertility history were recorded. All patients were also questioned regarding the use of pharmacological drugs such as glucocorticoid, due to the possibility of false negative results due to the inhibition of the corticotropic axis. There were no patients using this type of medication. Local ethics committee approval (Ref. No.: 71522473/050.01.04/42) was obtained from the Sakarya University Ethics Committee.

### 2.2 Auxiliary diagnostic methods

In the laboratory evaluation, serum total testosterone and DHEAS (dehydroepiandrosterone sulfate) levels were measured. Serum samples for laboratory examination were taken at the beginning of the cycle and early in the morning. The chemiluminescent microparticle immunotherapy (CMIA) method and radioimmunoassay (RIA) were used for DHEAS (Architect DHEA-S Kit; Abbott Architect I2000SR [autoanalyzer], Abbott Laboratories, Abbott Park, IL, USA) and total testosterone measurement (Architect 2nd Generation Testosterone Kit; Abbott Architect I2000SR [autoanalyzer, Abbott Laboratories, Illinois, IL, USA]), respectively. Additionally, transabdominal ultrasonographic examination was performed for evaluation of gynecological anatomical structures and possible PCOS diagnosis.

As the clinical auxiliary diagnostic method, the degree of hirsutism and menstrual patterns were evaluated. Oligomenorrhea was defined as having more than 35 days of menstrual intervals, while amenorrhea was defined as the condition of having no more than 6 months of menstruation consecutively. Hirsutism was determined according to the Modified Ferriman Gallwey Scoring System. Total scores of 8 or more on 9 different body surfaces obtained by inspection were defined as hirsutism.

All cases included in the study were grouped and compared according to the identified LOCAH (n = 16), PCOS (n = 122), and IH (n = 37) diagnoses. The Endocrine Society Clinical Practice Guidelines were used as a guide for the diagnosis of PCOS [14]. The absence of menstrual irregularity and lack of PCOS appearance on ultrasound was considered sufficient for the diagnosis of IH.

### 2.3 Definition of LOCAH

Basal 17-OH-progesterone levels of greater than 10 ng/mL were considered diagnostic criteria of LOCAH, while basal 17-OH-progesterone levels between 2–10 ng/mL were accepted as a mildly high limit. A high-dose (250 mcg) ACTH stimulation test was performed for all patients with basal values of 2–10 ng/mL. At 30, 60, 120, and 180 min after intramuscular ACTH injection, 17-OH-progesterone levels were measured from blood samples taken through a catheter placed intravenously. When any of these results were higher than 10 ng/mL, the result was interpreted as LOCAH. Genetic consultation for the confirmation of the genotyping of the CYP21A2 gene was recommended for all patients with test results greater than 10 ng/mL.

### 2.4 Statistical analysis

Data analysis was performed by using SPSS-22 for Windows (Statistical Package for Social Science, SPSS Inc., Chicago IL, USA). The variables were investigated using visual methods (histograms, probability plot) and analytical methods (Kolmogorov–Smirnov and Shapiro–Wilk tests) to determine whether or not they were normally distributed. We performed analyses to describe and summarize the distributions of variables. Continuous variables were reported as mean ± standard deviation and as whole number and percentages for categorical variables. One-way ANOVA test was used to compare multiple groups. One-way ANOVA followed by Duncan’s multiple comparison of the means revealed significant differences between the groups. We used the Kruskal–Wallis test to compare continuous nonparametric variables. The Mann–Whitney U test was performed for the double comparisons. The chi-square test (since the expected cell counts were sufficiently high) was used to compare the proportions in different groups. The statistically significant two-tailed P-value was determined to be <0.05.

## 3. Results

The mean values of modified FGS, total testosterone, DHEAS, and basal 17-OH-progesterone levels were 11.5 ± 3.5, 44.7 ± 15.5 ng/dL, 431.8 ± 153.0 mcg/dL, and 3.47 ± 1.16 ng/mL, respectively. Menstrual irregularity was present in 43.4% of all patients. Descriptive data are summarized in Table 1. According to the ACTH stimulation test result, 17-OH-progesterone response was higher than 10 ng/mL in 16 (9.1%) patients. In 4 patients, the response was over 15 ng/mL. The diagnosis was confirmed by genetic analysis in 9 of 16 patients. All patients with positive genetic results had a mutation in the CYP21A2 gene. The remaining 7 patients did not want to undergo genetic analysis or denied for economic reasons. According to all evaluation results, 16 (9.1%) of the patients were accepted as having LOCAH, 37 (21.1%) IH, and 122 (69.7%) PCOS.

**Table 1:** Baseline characteristics of patients.

Variables	Results*
Age, years	24.4 (±5.8)
Ferriman–Gallweyscore	11.5 (3.5)
Basal 17-OH-progesterone, ng/ml	3.47 (±1.16)
Total testosterone, ng/dL	44.7 (±15.5)
DHEAS , mcg/dL	431.8 (±153.0)
Menstrualirregularity, n (%)	76 (43.4)
FSH, IU/L	4.79 (±1.81)
LH, IU/L	5.89 (±4.80)
Oestradiol, pg/mL	46.9 (±51.8)
PRL, mcg/L	20.5 (±11.7)

SD: standard deviation, DHEAS: dehydroepiandrosterone sulfate, LOCAH: lateonsetcongenital adrenal hyperplasia, PCOS: polycystic ovary syndrome, FSH: follicle-stimulating hormone, LH: luteinizing hormone, PRL: prolactin. *Descriptive results for continuous variables were expressed as mean and standard deviation.

There was no difference between the groups in terms of average age. In the comparative analysis between the diagnostic groups, the mean DHEAS level was statistically different between the groups (P = 0.04) (Figure A). This difference was between LOCAH and PCOS according to post-hoc analysis (360.3 vs. 449.6, respectively; P = 0.044). There was no difference between the groups in terms of total testosterone levels (P = 0.461) (Figure B). When evaluated in terms of modified FGS, while there was no difference between LOCAH and PCOS (P = 0.146), IH was significantly lower than the other groups (P < 0.001) (Figure C). When basal 17-OH-progesterone levels were compared, the results were statistically significantly higher in LOCAH than in the other groups (P = 0.016) (Table 2). However, this difference was more pronounced between LOCAH and IH (4.70 vs. 3.17, respectively; P = 0.006).

**Figure:**
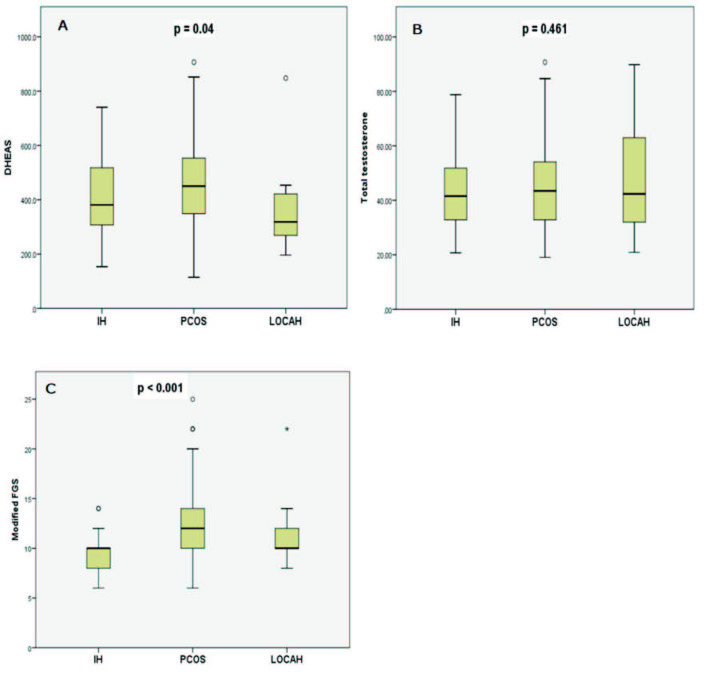
Summary of the comparison of DHEAS (A), total testosterone (B) and modified FGS (C) among the diagnostic groups.

**Table 2:** Summary of the comparison of age, DHEAS, total testosterone, basal 17-OH-progesterone, and Ferriman–Gallwey scores among the diagnostic groups.

Variables	Results*			P value
	LOCAH(n = 16)	PCOS(n = 122)	IH(n = 37)	
Age, years	26.1 (±6.9)	23.9 (±5.1)	25.2 (±7.3)	0.317
DHEAS, mcg/dL	360.3†† (±152.4)	449.6†† (±151.1)	403.9 (±150.0)	0.040
Total testosterone, ng/dL	47.2 (±20.5)	45.0 (±15.2)	42.7 (±14.4)	0.461
Basal 17-OH-progesterone, ng/mL	4.70† (±2.17)	3.40 (±0.96)	3.17 (±0.85)	0.016
Ferriman–Gallwey Score	11.1 (±3.3)	12.2 (±3.6)	9.2† (±2.1)	<0.001
Menstrual irregularity , n (%)	10 (62.5)	60 (49.6)	-†	<0.001

SD: standard deviation, DHEAS: dehydroepiandrosterone sulfate, LOCAH: lateonsetcongenital adrenal hyperplasia, PCOS: polycystic ovary syndrome, FSH: follicle-stimulating hormone, LH: luteinizing hormone, PRL: prolactin. *Descriptive results for continuous variables were expressed as mean and standard deviation. *Continuous variables were expressed as mean ± standard deviation, categorical variables were expressed as percentage and frequency. †This sign indicates that the difference is only within this group. ††There is a difference between the groups marked with this sign.

When compared in terms of menstrual irregularity, no statistically significant difference was found between LOCAH (62.5%) and PCOS (49.2%, P = 0.316). As expected, there were no menstrual irregularities in IH for diagnosis.

## 4. Discussion

In this study, we tested differential diagnosis for women with hirsutism with basal 17-OH-progesterone levels >2 ng/mL using serum DHEAS, serum testosterone levels, hirsutism score, and menstrual cycle characteristics without the need for an ACTH stimulation test. However, according to our results, laboratory features and clinical findings of patients with LOCAH were not particularly different from those of the other diagnoses.

Hirsutism is a clinical reflection of hyperandrogenism. Although the most common cause is PCOS, many reasons that may be benign or malignant can be considered in differential diagnosis [7,8]. In hirsutism, abnormalities of adrenal functions are found quite frequently. The clinical appearance of LOCAH, which develops due to enzyme deficiencies such as adrenal 21 alpha hydroxylase, may be indistinguishable from the clinical appearances of other forms of hyperandrogenemia, such as PCOS and IH [15]. Since the ovarian sonographic features and their responses to the same treatment are similar, the differential diagnosis of PCOS and LOCAH can be confused [16,17]. In some studies, patients with hirsutism diagnosed with IH and PCOS were diagnosed with LOCAH as a result of further examination [15]. Although LOCAH has a low incidence, the Endocrine Society Clinical Practice Guidelines recommends LOCAH screening for differential diagnosis in all patients with hyperandrogenemia [14].

In some ethnic groups such as Ashkenazi Jews, Mediterraneans, and Hispanics, the incidence of LOCAH in the general population can be observed at high rates of 1.9% to 3.7% [18]. Therefore, LOCAH should be considered in the differential diagnosis of hyperandrogenism in regions with these ethnic groups. In women with hyperandrogenemia, the prevalence of LOCAH is around 5% [8]. While genetic analysis is required for definitive diagnosis in adrenocortical enzyme deficiencies, measuring the basal 17-OH-progesterone level in the early follicular phase and performing the ACTH stimulation test if necessary are usually sufficient [13,19,20]. Although there is no fully accepted threshold value in adult women with hirsutism for the basal 17-OH-progesterone level, a level higher than 2 ng/mL is generally sufficient for diagnosis. However, values higher than this limit can overlap with both PCOS and LOCAH. In one study, basal 17-OH-progesterone levels of between 2–3 ng/mL were found in 19% and 25% of PCOS patients who were obese and lean, respectively [21]. In the current study, we selected only patients whose results were higher than 2ng/mL. Therefore, the role of other clinical and laboratory features in differential diagnosis is important before ACTH stimulation testing or genetic analysis is performed. Similarly, there is no generally accepted threshold for the 17-OH-progesterone response in the ACTH stimulation test. Even so, it provides a great deal of information in distinguishing LOCAH from a diagnosis of PCOS.

In women with hirsutism, DHEAS, total testosterone, modified FGS, and menstrual cycle query are routinely used for differential diagnosis. In adult women, 50%–60% of the circulating testosterone hormone is derived from the conversion of androstenedione in the liver under the action of 17b-hydroxysteroid dehydrogenase type 5 enzyme, while 25%–30% are from an ovarian source. The remaining 10%–15% of serum testosterone is produced directly from the adrenal cortex and released into the circulation [22,23]. In studies comparing LOCAH with other causes of hyperandrogenemia, higher testosterone levels have been reported in patients with LOCAH [7,24]. However, in one such study, it was reported that the mean of the modified FGS of all patients with hyperandrogenemia was between 16–18 and the baseline 17-OH-progesterone average in the LOCAH group was 14.9 ng/mL [21]. Compared to our study, it can be observed that our patients have lower clinical and laboratory results. Regardless, our total testosterone results were similar across all groups.

Under typical physiological conditions, 20%–30% of free DHEA in serum and almost all of DHEAs are of adrenal cortex origin [23]. Its production decreases with age, especially after the age of 30. The main mechanism that determines the amount in serum is 17,20 lyase (CYP17) enzyme activity [25]. In one study, 23 patients with LOCAH were compared with 27 healthy controls and 54 obese and 52 lean patients with PCOS, respectively. The mean of DHEAS in patients with LOCAH was 322 mcg/dL, and the results were 235 mcg/dL, 271 mcg/dL, and 248 mcg/dL in the compared groups, respectively [21]. Although the result was statistically significantly higher, the mean 17-OH-progesterone of LOCAH patients included in the study was recorded as 14.9 ng/mL. Additionally, the initial 17-OH-progesterone mean value of the patients with LOCAH in our study was 4.70 ng/mL, and the DHEAS level was surprisingly lower than that of the PCOS group. The reason for this result may be because the PCOS patients we selected had lower insulin levels (or insulin resistance), or the enzyme levels of our LOCAH patients were slightly better. In another study, the mean DHEAS of 18 patients with LOCAH was statistically higher than that of PCOS patients (348.5 vs. 235) [7]. However, information about basal 17-OH-progesterone levels was not obtained in this study. In addition, a negative correlation has been demonstrated between insulin resistance and DHEAS levels in PCOS patients [23]. This is explained by the fact that the activity of the enzyme CYP17 decreases with insulin resistance. Since high insulin resistance in PCOS is a very common feature, it reduces the usability of DHEAS level in differential diagnosis in such patients.

In the literature, oligoamenorrhea rates have been reported as between 52%–100% in PCOS patients and 23%–88.9% in LOCAH patients [15,23,24]. In our study, although the rate in PCOS patients was slightly lower than that in the literature, there was no significant difference between LOCAH and PCOS patients. Regardless of serum androgen levels, the hirsutism score was similar in LOCAH and PCOS patients, but lower in patients with IH than in the other groups. These results may indicate that the cases included in the study are not homogeneously distributed according to the clinical severity of their disease.

The current study has a number of limitations. First, there is a known negative correlation between DHEAS level and insulin resistance in PCOS. However, since we did not test insulin resistance in most patients, we could not evaluate this aspect. Second, we were not able to confirm all of our patients genetically. Therefore, ACTH stimulation test results were the main determinants for diagnosis.

In conclusion, the diagnosis of LOCAH is still a hormonally demanding process, as it requires genetic confirmation and counseling, especially in many patients whose enzyme activity is not very low. In this study, we investigated whether the diagnosis of LOCAH can be predicted by routine auxiliary tests and clinical findings without the need for the ACTH stimulation test. However, according to our results, although the auxiliary tests and clinical findings for the diagnosis of LOCAH contribute to the clinical interpretation, they are not superior to the use of the 17-OH-progesterone level for diagnosis.

## Informed Consent

This study was approved by the Local Ethics Committee of Sakarya University (11/02/2020, 71522473/050.01.04/42). Informed consent forms were signed by all patients. Data were obtained for scientific purposes.
